# The Role of Social Support in Cardiovascular Clinical Trial Participation among Black Men: Black Impact

**DOI:** 10.3390/ijerph191912041

**Published:** 2022-09-23

**Authors:** Sarah Addison, Yesol Yang, Faith Metlock, Mikayla King, Alicia McKoy, Amaris Williams, John Gregory, Darrell M. Gray, Joshua J. Joseph, Timiya S. Nolan

**Affiliations:** 1 College of Medicine, The Ohio State University, 370 W 9th Ave, Columbus, OH 43210, USA; 2 Comprehensive Cancer Center, The Ohio State University, 460 W 10th Ave, Columbus, OH 43210, USA; 3 College of Nursing, The Ohio State University, 1585 Neil Ave, Columbus, OH 43210, USA; 4 Center for Cancer Health Equity, The Ohio State University, 460 W 10th Ave, Columbus, OH 43210, USA; 5 National Center for Urban Solutions, The African American Male Wellness Agency, 2780 Airport Drive, Suite 333, Columbus, OH 43230, USA; 6 Anthem, Inc. (Formerly The Ohio State University College of Medicine), 1310 G Street, Washington, DC 20005, USA

**Keywords:** Black Impact, cardiovascular disease, Black men, social support, clinical trial participation, community-based participatory research

## Abstract

Background: Attainment of the American Heart Association’s Life’s Simple 7 (LS7) metrics reduces cardiovascular disease (CVD) risk; yet, Black Americans have the lowest LS7 attainment among all communities, the highest rate of CVD mortality, and low clinical trial participation. Social support is positively correlated with chronic disease self-management. Here, we describe the role of social support in a single-arm pilot clinical trial of a community-based lifestyle intervention among Black American men. Methods: The 24-week intervention featured weekly team-based physical activity and LS7-themed education. Seventy-four Black men participated in the intervention; twenty agreed to participate in exit surveys via one of three semi-structured focus groups. Data were transcribed verbatim and analyzed using content analysis framed by House’s social support framework. Results: Participants reported support from both peers and health coaches. The sub-themes of social support among peers were: (1) acknowledgement, understanding, and validation, (2) inspiration, (3) sense of community, (4) fear of disappointing fellow participants, and (5) group synergy. The sub-themes of social support from the health coaches and study team staff included: (1) contemplation of current health status, (2) racial concordance of health coaches and study team staff, (3) investment of the research team, (4) incentives, (5) access to healthcare providers, and (6) the COVID-19 pandemic. Emotional support was the most frequently discussed theme. Conclusions: Social support, especially emotional support, from peers and health coaches was a driver of clinical trial participation among participants. The intervention created a positive social environment and decreased medical mistrust. This intervention may provide a framework by which to facilitate clinical trial participation among Black men.

## 1. Introduction

Cardiovascular disease (CVD) remains the leading cause of death in the United States [1]. Among all races and ethnicities, Black Americans have the highest rate of CVD mortality in the United States [2]. In fact, the CVD mortality rate in Black American men is 32% higher than that of their White counterparts [1,3,4]. As the country becomes more diverse, addressing these disparate outcomes is vital to advance health equity.

The American Heart Association created Life’s Simple 7 (LS7), a seven-metric framework measuring cardiovascular health [1]. The metrics include physical activity, smoking cessation, optimal blood pressure, glucose and cholesterol, as well as maintaining a healthy weight and diet [5,6]. Understanding heart health along with increased attainment of these metrics can help patients reduce their risks of CVD, type 2 diabetes, and mortality [5,7,8,9,10]. However, Black Americans have the lowest attainment of Life Simple 7 among all racial/ethnic groups [11].

Despite this disproportionately high burden of CVD, Black Americans continue to be underrepresented in cardiovascular clinical trials [12,13,14]. As such trials have traditionally been on the forefront of medical advances, this imbalance in participation prevents generalizability and may exacerbate existing disparities. These inequalities can be mitigated by addressing social factors (e.g., social support, social networks, or social cohesion) that may influence the cardiovascular health of Black Americans [15,16,17]. Specifically, several studies have found a correlation between support provided by a social network (i.e., social support) and increasing physical activity, lowering blood pressure, as well as maintaining a healthy weight and diet [18,19].

Social support can improve participation in everyday activities that promote health [20,21,22,23]. For example, older adults with chronic disease showed greater satisfaction with participation in activities for health promotion when they received greater social support. This suggests that improvements in social support can have an impact upon satisfaction with participation, thus increasing their willingness to participate in health promotion activities. Social isolation and loneliness have long since been associated with increased rates of CVD, and mortality, and yet still little is understood about how specific components of social support impact CVD, or impact clinical trial participation among Black men [24]. As the gap in CVD-related mortality between White and Black men remains vast, it is imperative to investigate strategies to improve cardiovascular trial participation among Black men [3].

### Theory

To better understand which aspects of social support influence clinical trial participation, this study applied House’s social support theory. Per House, social support can be defined as the “perception and actuality that one is cared for, has assistance available from other people, and that one is part of a supportive social network” [25]. House’s Social Support Theory categorizes social support into four subtypes: “(1) appraisal (e.g., approval), (2) emotional (e.g., love, concern, understanding, reassurance, encouragement), (3) instrumental (e.g., aid, assistance), and (4) informational (e.g., advice, problem-solving information)” [25,26]. Under the guidance of House’s theory, this qualitative study aimed to identify and understand the types of social support men perceived during their participation in a targeted, community-based lifestyle change intervention, thereby providing evidence for future interventions that may lead to better cardiovascular health outcomes among Black men.

## 2. Methods

### 2.1. Study Design

The lifestyle change intervention, called Black Impact, aimed to improve attainment of LS7 in Black men. Participants in the Black Impact program (described in detail elsewhere [27]) underwent a 24-week, community-based lifestyle intervention adapted from the Diabetes Prevention Program and the American Heart Association’s Check, Change, Control program [28,29]. The program was implemented by health coaches (the study’s principal investigators) who were trained in the delivery of health education, medicine and/or advanced practice nursing: physicians (J.J.J., D.M.G.II.), nurse practitioner (T.S.N.) and personal trainers (trained to deliver a standardized physical activity program) who served as leads for each of six teams of participants. The teams were formed based on location and included a self-selected team leader whose responsibility was to send a weekly message to team members that encouraged the other team members to attend weekly sessions on a mutually agreed upon day and time at Parks and Recreation centers across the city. As a team, participants received 45 minutes of physical activity and 45 minutes of themed education during each session from the health coaches who all had previous experience in lifestyle change methods and facilitated the curriculum after the physical activity portion of the 24 weekly team sessions.

### 2.2. Participants and Recruitment

Black men who had poor or average cardiovascular health (< 4 LS7 metrics in the ideal range) at an annual walk/health fair were recruited into the single-arm pilot Black Impact program [27]. Eligible individuals were: (1) Black men (self-report); (2) adults aged 18 years or older; (3) poor or average cardiovascular health (< 4 LS7 metrics in the ideal range); (4) English speaking; (5) lived in Metropolitan Columbus, Ohio area; (6) no healthcare provider-imposed limitations on physical activity; and (7) willingness to participate in a group setting. Study investigators introduced the study to eligible individuals, followed by invitation to attend an informational session about the study. All participants gave written consent to participate in the Black Impact program; focus group participants gave additional verbal consent for the focus groups.

### 2.3. Data Collection

During the trial period, a self-administered battery of questionnaires and biometric assessments were completed at baseline, 12, and 24 weeks. Three months after the trial period, exit surveys were conducted in a subset of study participants engaging in focus groups to develop an in-depth, rich understanding of intervention feasibility. A single interviewer (T.S.N.) with two note takers (A.M. and F.M.) conducted three semi-structured, virtual focus groups (*n* = 20 participants) until data saturation was met. Each focus group was guided by an interview protocol to describe the characteristics of feasibility outlined by Bowen et al. [30] and explored intervention components such as perceptions of social support and clinical trial participation. Focus group responses were transcribed verbatim and reviewed for accuracy.

### 2.4. Analysis

Thematic analysis was performed as it is known to be a flexible method through which a detailed account of a sizable dataset can be provided [31,32]. For thematic data analysis, coders (T.S.N. [a qualitative methods expert], S.A., Y.Y., and M.K.) met to establish an a priori code list based on the interview protocol. Three coders (T.S.N., S.A., and M.K.) independently analyzed each transcript for significant statements, applying House’s framework, allowing for additions or subtractions of codes as necessary. All coders (T.S.N., S.A., Y.Y., and M.K.) met to iteratively reduce and refine codes until intercoder agreement was 100% and themes emerged. The following details qualitative findings around the social support Black men perceived during their clinical participation.

## 3. Results

### 3.1. Sample Characteristics

Twenty Black men participated in three focus groups. Black Impact participants who did vs. did not participate focus groups had similar age and sociodemographic characteristics (Table 1). The mean age of the men was 52 years. In total, 35% of focus group participants were married, 25% were divorced, and 35% were never married. Annual income ranged from < USD 20,000 (6%) to ≥ USD 75,000 (24%). In total, 80% were employed, 15% were retired, and 5% were unemployed. Among focus group participants, 70% had private insurance, 15% had Medicaid or Medicare, 5% had military insurance, and 10% had no insurance.

### 3.2. Types of Social Support and Source

Four types of social support were investigated using House’s social support theory. Appraisal support is defined as information from others that is useful for introspection, self-evaluation, or as constructive feedback, rather than information for the purpose of knowledge [25,33]. Emotional support is defined as love, trust, compassion, empathy, and a sense of belonging received from others. Instrumental support represents tangible items, tasks, or services performed or given by others. Finally, informational support involves suggestions, advice, and information gained by the recipient [25]. This is summarized in Table 2.

The thematic analysis revealed that men received support from two different sources: (1) peers and (2) researchers, the latter of which were healthcare providers who acted as health coaches during the intervention. Within these two sources, each of the four aspects of social support described by House was used to further code participant statements. Participants reported social support from peers in only two categories: appraisal and emotional support, with emotional support being the most mentioned (Table 3). Participants received all four types of social support from health coaches and study team staff (Table 4). Within this category, emotional support was also the most frequently cited.

### 3.3. Social Support from Peers

Two types of support, appraisal and emotional, were identified within House’s framework and will be discussed in this section. Appraisal support includes two sub-themes: (1) acknowledgement, understanding and validation, and (2) inspiration. Emotional support includes three sub-themes: (1) sense of community, (2) fear of disappointing fellow participants, and (3) group synergy.

#### 3.3.1. Appraisal Support 

##### Acknowledgment, Understanding and Validation

Black Impact participants engaged in weekly education and physical activity with one another during a time of social unrest and stark inequities in Black deaths related to the SARS-CoV-2 (COVID-19) pandemic. Participants remarked that participation in the Black Impact program afforded them the opportunity to reflect and find strength and comfort among a group of men with shared experiences. One participant stated, in reference to the other participants, “they’re going through the struggle with me… it lifted my spirits”. Some men spoke about how the group interactions enhanced their ability to cope with societal racism and discrimination. Specifically, a participant recounted, “just hearing other brothers’ reactions, responses to those instances of racism … let me know that I wasn’t alone in some of the feelings, and some of the things I was going through. That was real big, along with information.”

##### Inspiration

For some, the peer interactions within Black Impact brought them a sense of hopefulness. One individual in particular recounted being “really ashamed” at being unable to complete an exercise intervention, due to drinking the night before. However, he reported finding strength and solidary with others in the intervention also attempting to reduce the use of substances. He said, “[There was] another member of our team who was also doing that with his smoking habit, so [it] was kind of like supporting each other in that aspect. That was a big boost for my mental health, self-esteem, and confidence.”

#### 3.3.2. Emotional Support

##### Sense of Community

The participants spent 24 weeks in groups. Over time, participants noted that their “brotherhood” and “camaraderie” grew, and became a primary reason that they chose to continue with the study. To explain this sentiment around the “brotherhood”, participants stated that the presence and influence of the other participants provided “motivation” and “excitement”. Some participants said the camaraderie, aside from information on healthy lifestyle, was their primary takeaway from the intervention. One participant remarked that the weekly sessions became a “happy place”, while another commented: “I was going through a divorce, and I was like, I know I need village right now…… [the intervention was] so super vital to getting me through this whole last year [2020] … and the physical health outcomes was just icing on the cake”.

##### Connection between Participants, and the Fear of Disappointment

Additionally, study participants spoke of instances when they would have missed attending a session of the program but chose to attend because they did not want to “let their brother down.” Others felt their presence in the group was essential, and that their absence may affect the larger group. Others cited the camaraderie between peers as the primary factor holding them accountable, even when outside of the intervention, with one participant stating: “I got to make sure my numbers is good, because if I don’t then I’m letting down brother [name of participant]”. Some participants even took the initiative, unprompted by the research team, to contact members of the group who missed a session and encourage them to attend. One participant sent summaries of that week’s intervention and discussion topics to absent members. For other participants, the social interactions brought about through Black Impact were the catalyst for the formation of long-lasting friendships. Several of the men reported remaining in regular contact with their fellow participants, through Facebook groups, even after the intervention.

##### Group Synergy

Another stated the interactions with his peers had “pushed” him to “do more exercise.” Most participants also reported finding themselves “looking forward” to the weekly interventional sessions. Participants who found the initial physical activity sessions to be difficult, found solace in knowing other men were having the same challenges. In particular, one participant reported feeling “embarrassed” at the first exercise-based intervention but was put at ease knowing that his fellow participants, “the brothers”, were also “struggling”. Several men also recounted the impact of the relentless encouragement received from other peers in the intervention. One participant in particular went on to state: “We were always pushing each other, ‘you can do it’, ‘Come on, you can do this’ and then cheering each other on.” This team-based dynamic seemed to be a substantial contributory factor for several of the participants, with one individual explicitly stating: “One of the things that motivates me is just my connection with others and that whole team approach, so I was really looking forward to it, and it helped me.” The persistence of this team-based motivation is unknown; however, several men, even after the intervention, have made plans to go to the gym together, as a group.

### 3.4. Social Support from Health Coaches and Study Team Staff

All four types of social support were identified within House’s framework and will be discussed in this section. Appraisal support includes one sub-theme: (1) contemplation of current health status. Emotional support includes two sub-themes: (1) racial concordance of health coaches and study team staff, and (2) investment of research team. Instrumental support includes two sub-themes: (1) incentives, and (2) access to healthcare providers. Informational support includes one sub-theme: (1) SARS-CoV-2 pandemic.

#### 3.4.1. Appraisal Support

##### Contemplation of Current Health Status

The Black men participating in this study frequently spoke of experiencing a period of introspection regarding their health. Many described this period as when they began to understand the state of their health, for the first time. Some of the men reported realizing the false attribution of their health concerns to the aging process, but after going through the program, achieving quantitative results, and feeling healthier overall, came to an alternate conclusion. Notably, one man in particular stated that he came to realize his drinking habits were contrary to the philosophy of Black Impact. Another stated that the required weekly surveys made him contemplate the state of his health. Others remarked that merely seeing the metrics made them understand the severity of their current health status. Another reported that the intervention has made him want to take the initiative to follow up with his physician: “now that I’ve seen my numbers, I really literally want to go to my doctor.” Other members stated that they have renewed enthusiasm to address their long-term health and personal challenges thanks to the 24-week intervention, as well as the lasting community formed, with one participant stating: “I still have the same challenges that I faced, but it [the program] motivated me, and the brothers motivated me, to continue to do more.”

#### 3.4.2. Emotional Support

##### Racial Concordance of Health Coaches and Study Team Staff

Several men accredited their initial willingness to sign up for the intervention with having a team of researchers that “looked like me” or due to “the doctor being Black”. Notably, others spoke of their hesitancy in participating in a research study at The Ohio State University, a predominately White institution, being alleviated by the presence of a Black research team, with one man in particular stating: “we look at [The] Ohio State University…you got Black players that play on the football team, but when you look in the stands [it’s] White… [but] when you have Blacks actually speaking up on our behalf and coming to us with the information, it makes a major difference”.

Moreover, one participant in particular went on to express that he felt better “understood” by the program researchers, as they could better relate to his challenges as a Black man in the United States. Another recounted his experience in a prior clinical trial without Black representation. He is quoted as saying: “I mean some years ago… I was in a diabetes [intervention], it was looking at the effects of exercise and diabetes. Other than the coordinator, there was no one else who looked like me, and I just didn’t get the same type of energy from participating. It was more of a challenge and just wasn’t a good experience ultimately. So, this time around was much better.”

A few participants stated they were more likely to “believe” health reports when given from a Black researcher. “I take my information to a health coach, and I say, are these numbers good? She says, ‘oh you’re okay’, she’s giving it to me from a Black perspective and not from a White perspective”. Moreover, several men were also grateful for the regular unprompted check-ins provided by their study teams. “My blood pressure had dropped so low that I passed out… [A health coach], he just stayed with me, he helped me get through… he sat with me, [the] couple hours I was there, and [he] just really seemed to care about my health.”

##### Perceived Investment of Research Team

Across all focus groups, the men recounted the consistent attendance and enthusiasm of the study team, in regard to the team’s willingness to engage in workouts with the participants. Many reported this as having a profound and positive impact on them. “A health coach being there at 10 and a half months pregnant…… … and still working hard and helping us as much as [she] could. I was really impressed with [her], and [the other health coaches’] commitment was just out of this world… and that made me want to show up … [They] could have fluffed it off and did the bare minimum, but [they] even got up and worked out with us”. Others pointed out and appreciated the health coaches’ dedication to the men, despite “busy” schedules, with a participant stating: “I know you guys have had your personal lives and you would come to these workouts after having a whole workday”.

#### 3.4.3. Instrumental Support

##### Incentives

At the intervention’s onset, all participants were given exercise supplies, such as Garmin watches, resistance bands, and athletic shoes. Many of the men reported using these supplies, even after the program’s conclusion. Others were particularly grateful for these supplies, as they served as instruments to keep the men active at home during the 2020 lockdown caused by the COVID-19 pandemic, in which many gyms were inaccessible. One participant stated: “some of the things that they gave us to help us through this like the [Garmins], the shoes, the workout bands…I think those are all great things to give to us as tools.”

Other incentives, such as gift cards, were offered, to encourage participants to fill out surveys. A few of the men remarked that these gift cards made them feel as though the study team valued their time. Others reported enjoying the “perk” of gift cards and felt motivated to complete the surveys as a result. One participant specifically stated: “Something else that I liked was the fact that, with many of these questionnaires and information seeking things that were done…you treated us with gift cards, and I’m always about a gift card”.

##### Access to Healthcare Providers

Study participants reported feeling sincerely cared for the by the study team’s staff, physicians, and nurse practitioner, due to their willingness to spend time with them, address their personal cardiovascular health metrics, and reach out directly to their personal doctors. “One thing that stuck out for me was [health coach] when my BP numbers [weren’t going] down… he pulled me to the side… and asked for my doctor’s information. He actually took time out of his busy day to reach out to my doctor.”

#### 3.4.4. Informational Support

##### SARS-Cov-2 (COVID-19) Pandemic

Several participants reported feeling relieved and reassured by the ability to receive information on the COVID-19 pandemic in “real time”, by a team of researchers and providers they trusted, a feeling that was only amplified in the face of rampant misinformation. The study team was also able to ease the trepidation felt by many participants surrounding COVID-19 vaccination. Notably, a participant stated that his hesitancy to receive the COVID-19 vaccine was dispelled after an in-depth discussion with a health coach. Others reported similar experiences, with one in particular stating: “[A health coach] was very instrumental in me getting the vaccine, because I was kind of on the fence about it. [He] made me feel better about going ahead and getting it. So, I went ahead and got both vaccines. “After a discussion with a health coach, another participant reported not only getting vaccinated, but also feeling comfortable and empowered to encourage others to do the same. Similarly, another man reported that despite his initial hesitancy in receiving the vaccine, his fears dissipated when he watched a video of a health coach receiving the vaccine.

## 4. Discussion

To our knowledge, this is the first study to describe the role of social support in clinical trial participation among Black men. Our study was co-designed using the principles of CBPR that intentionally engage participants and stakeholders. In accordance with a program to improve LS7, this approach has not been adequately studied in Black men [34]. Participants garnered substantial amounts of support from both their intervention peers and the research team. Further, emotional support seemed to be the foundation for increased trust in the healthcare system, as well as the sense of community experienced by the men. These results are of timely importance, considering we are approaching the thirty-year anniversary of the 1993 NIH Revitalization Act, and yet still, we remain no closer to adequate representation of Black Americans, as was demonstrated in a recent study revealing a mere 2.9% participation rate of Black people in recent cardiovascular drug trials [12,35]. Over the years, several strategies to improve participation among Black Americans have been proposed, with varying degrees of success [36,37]. Fostering social support among participants and researchers may be another avenue through which participation can be bolstered.

### 4.1. Peer Support

The relationship between peers had a positive impact on the participants’ mental health. Amidst the stress of racial turmoil, the men (peers) were able to lean on one another and validate each other’s experiences as a Black man in society. Support from peers has been associated with a reduction in perceived stress among Black men, and this study adds to the evidence supporting this finding [38]. Several men from this cohort reported feeling a sense of relief in hearing that other participants were having similar personal, professional, and physical challenges. For many Black men, such conversations are traditionally considered off-limits; however, the bond formed among the participants appeared to facilitate these interactions [39].

The men spoke profusely of the “brotherhood” formed among participants, life as a Black man in America, and the sense of belonging that formed as a result. Their bond expanded so much that some took it upon themselves to update absent members on the intervention’s progression and meet outside of the intervention. Several men spoke about the influence of the brotherhood on their retention and their adherence to the intervention. The men expressed that the brotherhood was particularly important during the COVID-19 pandemic and racial unrest of 2020. The social isolation, as well as emotional turmoil, appeared to be an additional factor bonding them together. For many within the cohort, this aspect of emotional and appraisal social support lessened the burden of discrimination they felt within this distressing time. Perceived racial discrimination has been known to be mitigated by high levels of emotional support [40], with studies demonstrating stronger effects of discrimination among those with lower levels of social support [40,41]. Moreover, recent studies have described the detrimental effects of discrimination on physical health, suggesting that social support may be a means to lessen the effects of discrimination on physical health, although more studies must be done to determine if an association truly exists [42,43].

Throughout the intervention, the men stated they persisted onwards due to their “team” or felt that they were letting down teammates by not showing up. These findings align with other studies, which also found that peer and/or friend support are facilitating factors in physical activity [44,45,46,47] and medication/treatment adherence [48,49,50,51]. This influence on patient morale is consistent with our results, suggesting that social support may have positive effects on CVD health in Black men. These findings demonstrate that cultivating strong bonds among intervention participants may be an avenue through which researchers may increase intervention effects and retention.

### 4.2. Support from Health Coaches and Study Team Staff

Several men expressed that the Black Impact intervention was the first time they truly understood the importance of consistent healthcare, and/or that they were unhealthy. Over the course of the 24-week intervention, many study participants transitioned from the pre-contemplation stage of change to action. This transition is vital, as a significant change in behavior is notoriously difficult to achieve in the precontemplation stage [52,53]. This would not have been possible without the trust-building that occurred through interactions with the research team. Further, as the continuity of care by healthcare providers has been associated with reduced mortality rates, each Black Impact participant was connected to a racially concordant primary care provider, or a provider who had completed implicit bias training [54].

Across all focus groups, the men spoke highly of the bond formed with the staff, and the investment of the research team’s health coaches. Several men described negative past experiences with healthcare providers that amplified their wariness of the healthcare system. However, by the intervention’s conclusion, many held a more positive view of healthcare providers and medical researchers, along with a greater sense of trust. According to participants, the formation of the relationship between the research team and the men had a remarkably positive impact on their motivation to participate and adhere to the intervention, as well as their openness to participating in future clinical trials. This finding is congruent with a review study conducted by Otado and colleagues, which revealed that “principal investigators and study coordinators cultivating rapport with participants” was among the most successful retention strategies for clinical trials focused on retaining Black Americans [36].

In our study, the relationship between the health coaches and participants flourished through the existing connection of the community to the African American Male Wellness Agency (AAMWA). For many of the men, this connection to a long-standing community stakeholder laid the foundation on which researcher–participant rapport was built. Our team modeled these interactions in its application of community-based participatory research (CBPR), which has been used to build relationships between communities and researchers [34,55,56]. This connection was further fostered through the research team’s willingness to involve the participants in the research process as equal partners, as well as the research team’s dedication. Notably, many of the men mentioned that the team’s health coaches worked out with them even if it was late at night, rain or shine, and also made time for them outside of the intervention. Several of the men expressed that through these experiences, they felt genuinely cared for by the research team.

For many, the racial concordance of the health coaches and study staff was also a major facilitating factor. Several of the men stated feeling more able to rely on information from the research team, because they too understood what it is like to be Black in America. Through shared cultural experiences, the participants were able to bond with the research team. While racial concordance in research teams may not always be possible, it is important to note that that racial concordance is merely an entry way into the development of a deeper relationship with participants and/or patients, and interpersonal relationships between participants and researchers/providers can be fostered when research teams are not racially concordant [36,57,58]. Diversity and inclusion in future research teams are feasible for future clinical trials, but at the very least non-racially concordant researchers can prioritize approaching participants with cultural humility and purposeful transparency to build trust and rapport [59,60,61].

Surprisingly, the majority of instrumental support the participants spoke of was not represented by the incentives provided but was rather access to healthcare providers. Repeatedly, the men noted the importance of being able to contact the research team’s providers on a regular basis. Across focus groups, many men spoke glowingly of the care provided. For many, the racial concordance of the providers was again a large factor. These results are in line with recent studies demonstrating that racial concordance between Black patients and their providers improved communication, preventive care and rates of immunizations, and decreased total healthcare expenditures [62,63]. This further underscores the need for increased levels of communication and transparency between Black patients and their non-racially concordant providers. Regarding the tangible support provided to the men, the gift cards appeared to encourage their participation in end-surveys. The supplies provided also encouraged participants to engage in home-based workouts amidst mass lockdowns during the height of the COVID-19 pandemic.

Across all focus groups, the men recounted a dramatic increase in their knowledge of CVD and spoke positively of the education they received on exercise, healthy eating, and other metrics involved in CVD, a finding consistent with other CVD interventions [64]. Whether decreased knowledge of CVD and its risk factors is correlated with poorer outcomes remains unclear; however, educating participants should always be a priority in interventions [65,66]. The men attributed the education they received to increasing their confidence in taking ownership of their personal health. Although the education received by participants was a cross-cutting theme, due to the timing of the intervention, the communication about the COVID-19 pandemic was the predominant aspect of informational social support the men spoke of. These results suggest that discussions with a trusted healthcare professional around health promotion and current events can be perceived as beneficial to Black men.

### 4.3. Limitations

This study has four primary limitations. First, by its very nature, qualitative analysis cannot be used to draw conclusions, but only to make inferences. Thus, it is difficult to determine whether these findings can be generalized to other subsets of participants. Secondly, the focus group participants were not significantly different in terms of demographics, education, or socioeconomic status from Black Impact participants who did not take part in the focus groups. However, it is possible that alternate intervention participants may have brought forth differing opinions and inferences on the role of social support in their experience. Next, participants were recruited from a health-related event, so it is feasible that such individuals may be more invested in improving their health when compared to the general population. However, the criteria to participate in the study included less than ideal cardiovascular health, suggesting that participants were not engaged in effective strategies to support their health. Finally, all coders of the data were Black women. This homogeneity among analysts may have skewed the interpretation of the results. However, such homogeneity is not unusual among writers of scientific papers, and great care was taken to reduce any potential bias in the coding process.

## 5. Conclusions

This study examined the role of social support in a cardiovascular health clinical trial, focused on Black American men. Social support, especially emotional support, from peers and health coaches was a primary driver of participation in the Black Impact trial. Not only did the intervention improve LS7 attainment, but it also created a positive social environment and decreased medical mistrust among the men. Our results highlight the need for transparency, cultural humility, and the facilitation of rapport-building between intervention participants, as well as between participants and researchers. This may provide a framework to facilitate clinical trial participation and improve intervention effects among Black men.

## Figures and Tables

**Table 1:** Age and Sociodemographic Characteristics of Focus Group Participants vs. Non-Focus Group Participants in Black Impact.

	No Focus Group (*N* = 54)	Focus Group (*N* = 20)	Overall (*N* = 74)	*p*-Value
**Age**	51.8 (10.5)	53.3 (10.3)	52.2 (10.4)	0.583
**Marital Status**				0.169
Married	31 (57.4%)	7 (35%)	38 (51.4%)	
Widowed	0 (0%)	1 (5%)	1 (1.4%)	
Divorced	8 (14.8%)	5 (25%)	13 (17.6%)	
Separated	1 (1.9%)	0 (0%)	1 (1.4%)	
Never Married	11 (20.4%)	7 (35%)	18 (24.3%)	
Missing	3 (5.6%)	0 (0%)	3 (4.1%)	
**Annual Income**				0.682
<USD 20,000	3 (5.6%)	2 (10%)	5 (6.8%)	
USD 20,000–49,999	13 (24.1%)	7 (35%)	20 (27%)	
USD 50,000–74,999	16 (29.6%)	6 (30%)	22 (29.7%)	
≥USD 75000	13 (24.1%)	3 (15%)	16 (21.6%)	
Missing	9 (16.7%)	2 (10%)	11 (14.9%)	
**Employment Status**				0.612
Employed	44 (81.5%)	16 (80%)	60 (81.1%)	
Retired	4 (7.4%)	3 (15%)	7 (9.5%)	
Unemployed	4 (7.4%)	1 (5%)	5 (6.8%)	
Missing	2 (3.7%)	0 (0%)	2 (2.7%)	
**Health Insurance**				0.908
Private insurance	38 (70.4%)	14 (70%)	52 (70.3%)	
Medicaid/Medicare	5 (9.3%)	3 (15%)	8 (10.8%)	
Military insurance	3 (5.6%)	1 (5%)	4 (5.4%)	
No insurance	7 (13%)	2 (10%)	9 (12.2%)	
Missing	1 (1.9%)	0 (0%)	1 (1.4%)	

Legend: numbers are mean (standard deviation) or count (percentage). *p*-Values were calculated using *t*-tests or X2 tests, where appropriate.

**Table 2:** Types of Social Support and Codebook Descriptions [25].

Types of Social Support	
Type of Support	Codebook Description of Type of Support
Appraisal	Information from others that is useful for introspection, self-evaluation, or as constructive feedback, rather than information for the purpose of knowledge.
Emotional	Love, trust, compassion, empathy, and a sense of belonging received from others.
Instrumental	Tangible items, tasks, or services performed by or given by others.
Informational	Suggestions, advice, and information gained by the recipient.

**Table 3:** Peer Support.

Peer Support.		
Major Theme	Sub-Theme	Codebook Description of Type of Support
Appraisal	Mental Health	“I still have the same challenges that I faced, but [the program] motivated me, and the brothers motivated me, to continue to do more.” “I was going through a divorce, and I was like, I know I need village right now more than ever. You know [what] I’m saying? Not that I used it as a support group, and came and boo-hooed or anything like that, but just being around the positive energy every week, really kept the wind in my back… and so super vital to get me getting me through this whole last year … and the health outcomes, the physical health outcomes were just icing on the cake for me. It was the emotional health outcomes and the mental health outcomes that took [the program] to another level.”
Emotional	Sense of Brotherhood	“I don’t know what would [have] kept me in besides that (brotherhood).” “It just reinforces what you already know about your fellow brother and your fellow man…give them the opportunity that we can do better, and there are many out there that are doing better, but just the fact that you have that brotherhood and you have that relationship with people that you see in your communities.”

**Table 4:** Clinical Support.

Clinical Support		
Major Theme	Sub-Theme	Codebook Description of Type of Support
Instrumental	Incentive	“You know the little gift cards were good sometimes as an incentive. Those little things that were beneficial.” “[I] believe that the Amazon cards incentivized us as well. I think that’s a good way because you’re not doing it for nothing. You know here’s something for your time. I think that was a good gesture.”
	Perceived Investment of Study Team	“One thing that stuck out for me was [D.M.G.] when my BP numbers wouldn’t go down … he pulled me to the side … and asked for my doctor’s information. He actually took time out of his busy day to reach out to my doctor.” “My blood pressure had dropped so low that I passed out and ended up going to emergency [room], and Dr. Josh (J.J.J.) just stayed with me. He helped me get through so I could kind of get through faster.”
Appraisal	Health	“But going through the program certainly kicked up my activity level, which I think helped [me] break through some of the plateaus and barriers that I was already experiencing. [I] just needed another reason a little more of a push… a little more mindful eating and stuff. So, I think that from a health perspective it helped reinforce some of the things that I knew. And it helped dispel some of the things I didn’t know. So, yeah it was overall it was definitely a good supplementation event to participate in for me.” “I’m being more self-conscious now with my eating more fruits and vegetables. Doing more protein smoothie shakes in the morning for breakfast. More water intake so slowly but surely I’m doing the things that I need to do that I should have done, I really think the program because the program really helped me push me into that mindset, to do more…”
Informational	Knowledge of COVID-19	“Dr. Joseph (J.J.J.) was very instrumental in me getting the vaccine because I was kind of on the fence about it. And he kind of made me feel better about going ahead and getting it. So, I went ahead and got both vaccines.” “And I will share that the feedback from the doctors about the vaccine [and] about how they felt about the vaccines helped boost my confidence in taking the vaccine. So I’m good now.”
	Access to Provider	“As far as this program is concerned, I never had that many young black urban professionals that tended to my personal needs if you guys knew how that made me feel … I was so proud. Dr. Gray (D.M.G.II.) was the main one on our text checking in keeping up with everybody just checking in and say how y’all doing everything cool?” “I had access to him (J.J.J.), so I think that was one of the things that that I really appreciate it and enjoyed about the program just the information the information and the access.”
Emotional	Influences of Black Researchers	“I mean some years ago … I was in a diabetes [intervention]. It was looking at the effects of exercise and diabetes. Other than the coordinator, there was no one else who looked like me, and I just didn’t get the same type of energy from participating. It was more of a challenge and just wasn’t a good experience ultimately. So this time around was much better.” “You understand some of the struggles and things we go through, and its much easier to relate to [health coaches], because [they] relate to us.”
	Perceived Investment of Study Team	“My blood pressure had dropped so low that I passed out … Dr. Josh (J.J.J.) he just stayed with me, he helped me get through … he sat with me for a couple hours. I was there, and (J.J.J.) just really seemed to care about my health.” “Dr. Nolan (T.S.N.) being there at 10 and a half weeks pregnant … and still working hard and helping us as much as [she] could. I was really impressed with [her], and [the other health coaches’] commitment was just out of this world… and that made me want to show up … [They] could have fluffed it off and did the bare minimum, but [they] even got up and worked out with us.”

## Data Availability

Data available on request due to restrictions eg privacy or ethical. Because of the sensitive nature of the data collected for this study, access to the data set from researchers trained in human subject confidentiality protocols may be requested from the corresponding author.
